# Enhanced sentinel surveillance for hepatitis B infection in 200 counties in China, 2013-2016

**DOI:** 10.1371/journal.pone.0215580

**Published:** 2019-04-23

**Authors:** Ning Miao, Hui Zheng, Xiaojin Sun, Liping Shen, Feng Wang, Fuqiang Cui, Zundong Yin, Guomin Zhang, Fuzhen Wang

**Affiliations:** 1 Chinese Center for Disease Control and Prevention, Beijing,China; 2 School of Public Health, Peking University, Beijing, China; The University of Hong Kong, CHINA

## Abstract

Hepatitis B infection is a major public health challenge in China. Clinicians report hepatitis B cases to the National Notifiable Disease Reporting System. A 2007 study found that only 35% of hepatitis B cases that had been reported as acute infections met a rigorous case definition of acute hepatitis B, implying overreporting of new-onset infections. To increase the accuracy of reported acute hepatitis B infections, in 2013, we initiated enhanced hepatitis B surveillance in 200 sentinel counties. We compared incidences and proportions of different stages of hepatitis B infection before and after implementation of enhanced surveillance. We checked the accuracy of reported data and re-diagnosed hepatitis B cases reported as acute infection according to the enhanced diagnostic criteria and calculated positive predictive value(PPV) of acute hepatitis B reports. Compared to previous surveillance, with enhanced surveillance, the incidence of reported acute hepatitis B infection decreased by 53.7% and the proportion of unclassified hepatitis B infection was reduced by 79.4%. From 2013 to 2016, the PPV of acute hepatitis B increased (55.8% to 71.0%); PPV rates in western and rural areas were lower than in other areas. We recommend enhancing hepatitis B surveillance nationwide using these new standards, and raising western and rural areas clinicians’ diagnostic and reporting capacity, and ensuring sufficient resources for IgM anti-HBc testing.

## Introduction

An estimated 291 million people were living with chronic Hepatitis B Virus (HBV) infection around the world in 2016. HBV has been highly endemic in China, as historical HBV transmission built a reservoir of approximately 86 million chronically infected persons, accounting for 30% of the global burden of chronic HBV infection[1].

Viral hepatitis cases have been reported to China’s National Notifiable Disease Reporting System (NNDRS) since 1959, and detailed virus subtyping has been available since 1990. Starting in 2004, cases are being entered into an online NNDRS database by diagnostic hospitals, [2, 3] so that now, hepatitis B surveillance is a hospital-based, passive national surveillance system that enables all healthcare institutions to report hepatitis B cases[4]. Both acute and chronic hepatitis B (AHB and CHB) cases are reported, and cases that cannot be clearly diagnosed are indicated as unclassified cases in the system. Case information, including name, age, sex, occupation, birthdate, location of residence, classification of cases, and date of onset is available in NNDRS.

Although the online reporting system has significantly increased the efficiency of reporting (over 10 million hepatitis B cases have been reported since 2004 [5]), previous study has shown that only 35% of reported AHB cases were verifiable, acute hepatitis B infections based on IgM anti-HBc testing and patients’ histories of hepatitis B infection [6]. This may in part be because IgM anti-HBc testing was available in fewer than 1.5% of reporting hospitals, affecting diagnostic specificity[7].

In 2013, an enhanced sentinel surveillance system was established in 200 counties in China to improve the accuracy of reported AHB and the epidemiology of hepatitis B infection. We report the impact of enhanced surveillance on the apparent epidemiology of HBV infection and the accuracy of acute hepatitis B reports to NNDRS.

## Methods

### Setting

China CDC established a hepatitis B sentinel surveillance system in 2013 in 200 counties of the 31 provinces in China; half of the counties were urban according to the National Statistical Bureau’s definition (http://www.stats.gov.cn/tjsj/tjbz/tjyqhdmhcxhfdm/, accessed January 10, 2019). Population demographics and previous reported hepatitis B case counts vary substantially by county, and to maximize the sentinel site population size and number of case reports, selected sentinel counties met both of the following criteria, (1) population greater than 300,000, and (2) number of hepatitis B cases reported in 2012 of at least 200.

**Fig 1:**
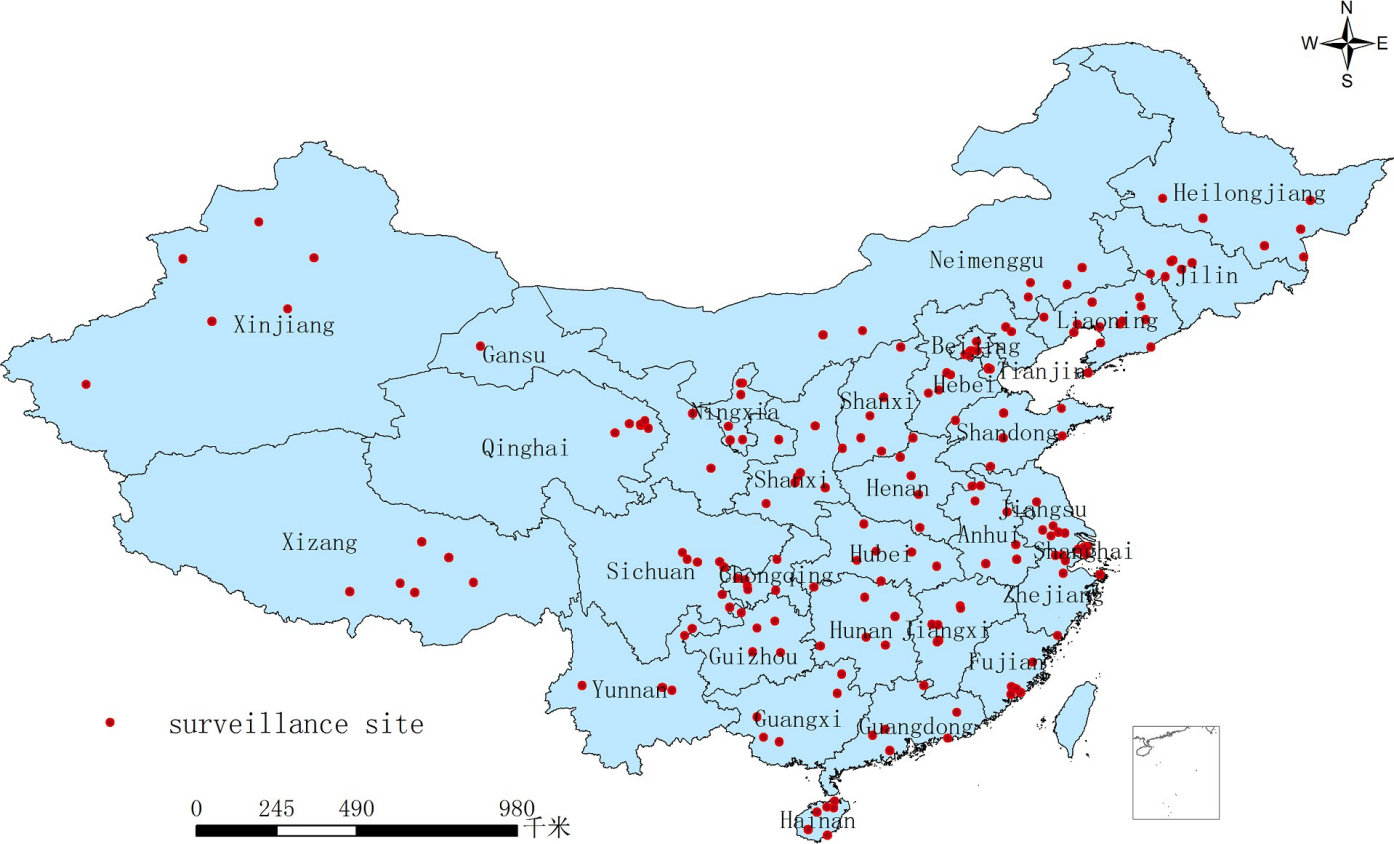
200 surveillance sites for hepatitis B in China. Fig 1 represents 200 surveillance sites for hepatitis B in China. Each province has a hepatitis B surveillance site. A red dot represents a surveillance county.

Regions were defined as eastern (Beijing, Tianjin, Hebei, Liaoning, Fujian, Guangdong, Jiangsu, Shandong, Shanghai, Zhejiang and Hainan), central (Anhui, Heilongjiang, Henan, Hubei, Hunan, Jiangxi, Jilin, and Shanxi), and western (Chongqing, Gansu, Guangxi, Guizhou, Inner Mongolia, Ningxia, Qinghai, Shaanxi, Sichuan, Tibet, Yunnan, and Xinjiang). Fig 1 shows a map indicating locations of the selected sites.

### Design, enhanced surveillance, and data sources

This was an observational study comparing NNDRS data before (2009–2012) and after (2013–2016) the 2013 enhancement of hepatitis B infection surveillance in these 200 sentinel counties. We compared changes in the apparent epidemiology of hepatitis B infection before-and-after surveillance enhancement, and we described AHB PPV after surveillance enhancement (2013–2016).

**Fig 2:**
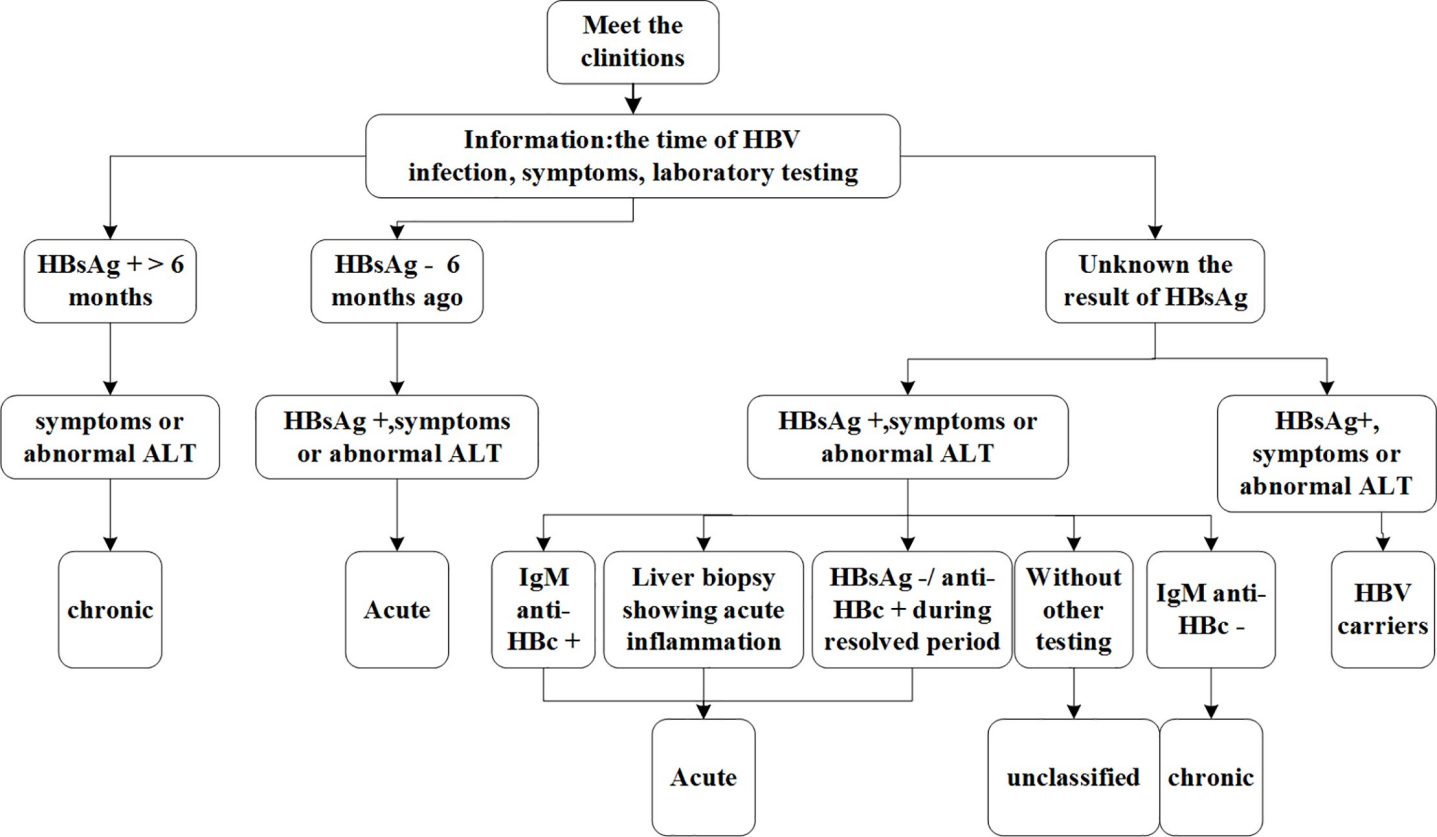
Standardized diagnosis and reporting process in surveillance point, China. Fig 2 represents standardized the diagnostic and reporting procedures for clinicians based on national diagnostic criteria. This flowchart reminds clinicians how to diagnose acute and chronic hepatitis B.

Enhanced surveillance was (1) standardizing the diagnostic and reporting procedures for clinicians based on national diagnostic criteria, summarized in Fig 2, (2) adding fields to NNDRS for additional information helpful for the diagnosis the stage of hepatitis B infection, including the date of initial HBsAg positivity, hepatitis B signs and symptoms, ALT and IgM antibody to hepatitis B core antigen (IgM anti-HBc) laboratory results, liver biopsy status, changes in HBsAg and Anti-HBs serology during a resolving infection period, and (3) obtaining addition serum from patients with suspected AHB for re-testing by the China CDC national laboratory for IgM anti-HBc.

We obtained data from NNDRS on case reports of AHB, CHB, and unclassified hepatitis B (UHB) that were reported by hospitals in these sentinel surveillance counties between January 1, 2009 and December 31, 2016.

### Laboratory testing

Three milliliters of venous blood was obtained by the clinicians from AHB cases they were reporting to NNDRS and sent to National Hepatitis Laboratory of the Institute for Viral Disease Control and Prevention, China CDC. Sera were stored at–30C before being tested. All serum specimens were tested for anti-HBc IgM using Abbott microparticle enzyme immunoassay reagents (Abbott Laboratories, Chicago, IL, USA). Specimens yielding inconsistent or indeterminate results were retested. ALT tests were performed by the hospital laboratories, and were not repeated at the national laboratory.

### Diagnostic case review

We re-diagnosed all AHB cases reported after enhancement of surveillance (2013–2016) using the national diagnostic criteria for hepatitis B (WS299-2008) [8] combined with the additional NNDRS data from the enhanced surveillance. Cases were considered AHB if the met at least one of the following two criteria: (1) HBsAg positive, with discrete onset of symptoms (e.g. nausea, vomiting, diarrhea, anorexia, abdominal pain, and jaundice), and IgM anti-HBc positive, (2) HBsAg positive, with abnormal ALT levels (>40 IU/m) and IgM anti-HBc positive.

### Statistical methods

Incidence rates were analyzed by time period (before and after enhancement of surveillance), age group, gender, and region of the country. The positive predictive value(PPV) for diagnosis of AHB [9] was defined as the proportion of reported AHB cases that were re-diagnosed as confirmed, acute hepatitis B infection using the nationally-standardized definitions. Chi-square and trend chi-square tests were performed using SPSS 23.0 software. P <0.05 was considered as statistically significant.

### Ethical review

This observational study was considered routine work to improve notifiable disease surveillance, and did not require additional Ethical Review Committee approval. Individuals were not identifiable in NNDRS data used in this study.

## Results

### Hepatitis B incidence

**Fig 3:**
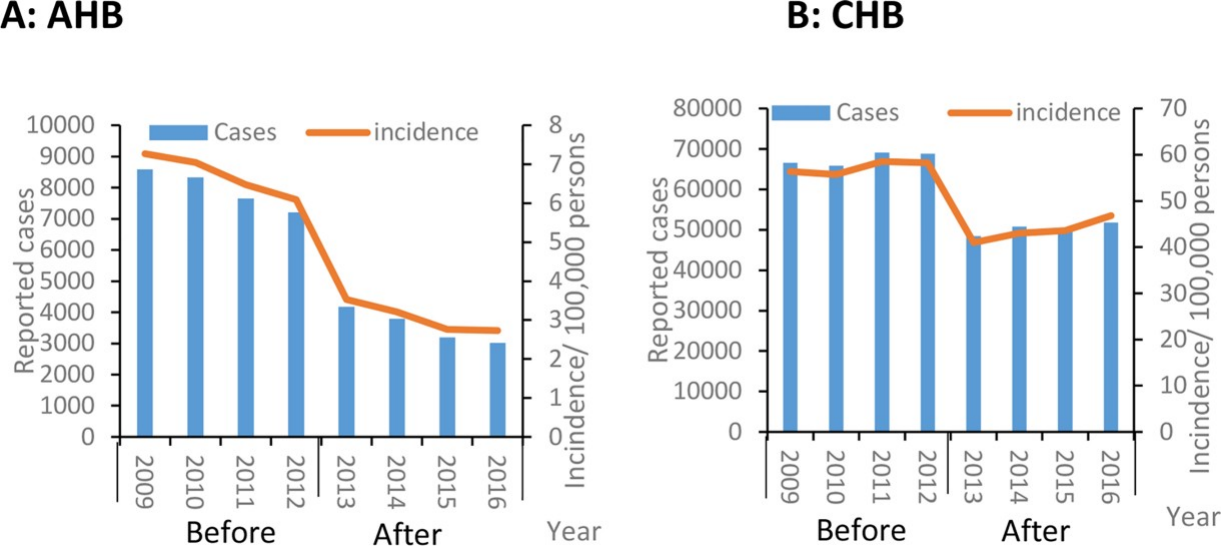
Reported cases and incidence of hepatitis B (acute and chronic) before and after sentinel surveillance, China, 2009–2016. Reported cases and incidence of acute hepatitis B. (B) Reported cases and incidence of chronic hepatitis B.

In the four pre-enhanced surveillance years (2009 to 2012), 31,769 AHB cases were reported; in the four post-enhanced surveillance years (2013–2016), 14,176 AHB cases were reported. More than 7,000 AHB cases were reported annually to NNDRS before enhanced surveillance, compared with an average of 3,544 in 2013 through 2016. The annual AHB reported incidence declined from 6.7/100,000 before enhanced surveillance to 3.1/100,000 after enhancement of surveillance—a decrease of 53.7% (Fig 3).

Fig 3 shows the incidence of CHB, which ranged from 48,416 to 51,784 after enhanced surveillance compared with before enhanced surveillance (65,820 to 69,132). The incidence of CHB declined by 23.8% after surveillance enhancement: 57.2/100,000 to 43.6/100,000.

**Fig 4:**
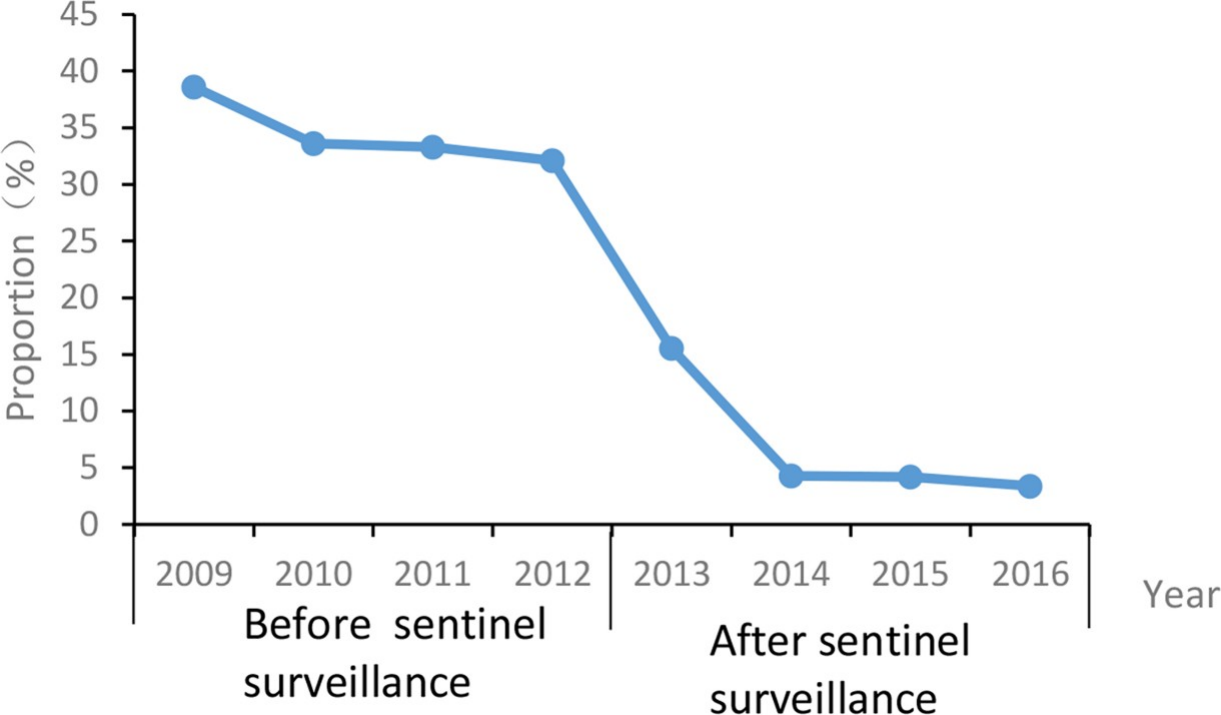
The proportion of unclassified hepatitis B, China, before and after sentinel surveillance. Fig 4 represents the proportion of unclassified hepatitis B, China, before and after sentinel surveillance. The proportion of unclassified hepatitis B is equal to the number of unclassified hepatitis B divided by the total number of hepatitis B cases.

Before enhanced surveillance, 159,103 UHB cases were reported; after enhanced sentinel surveillance 16,390 UHB cases were reported. Before enhanced surveillance, the annual proportion of UHB cases among all reported cases of hepatitis B infection varied little between 2009 and 2012 (38.6% to 32.1%, mean 34.5%; trend χ2 = 0.98, P >0.05). After enhancing surveillance, the annual proportion of UHB cases decreased from 15.6% in 2013 to 3.4% in 2016. (trend χ2 = 11.04, P <0.0001) (See Fig 4).

### Incidence by age and gender

**Fig 5:**
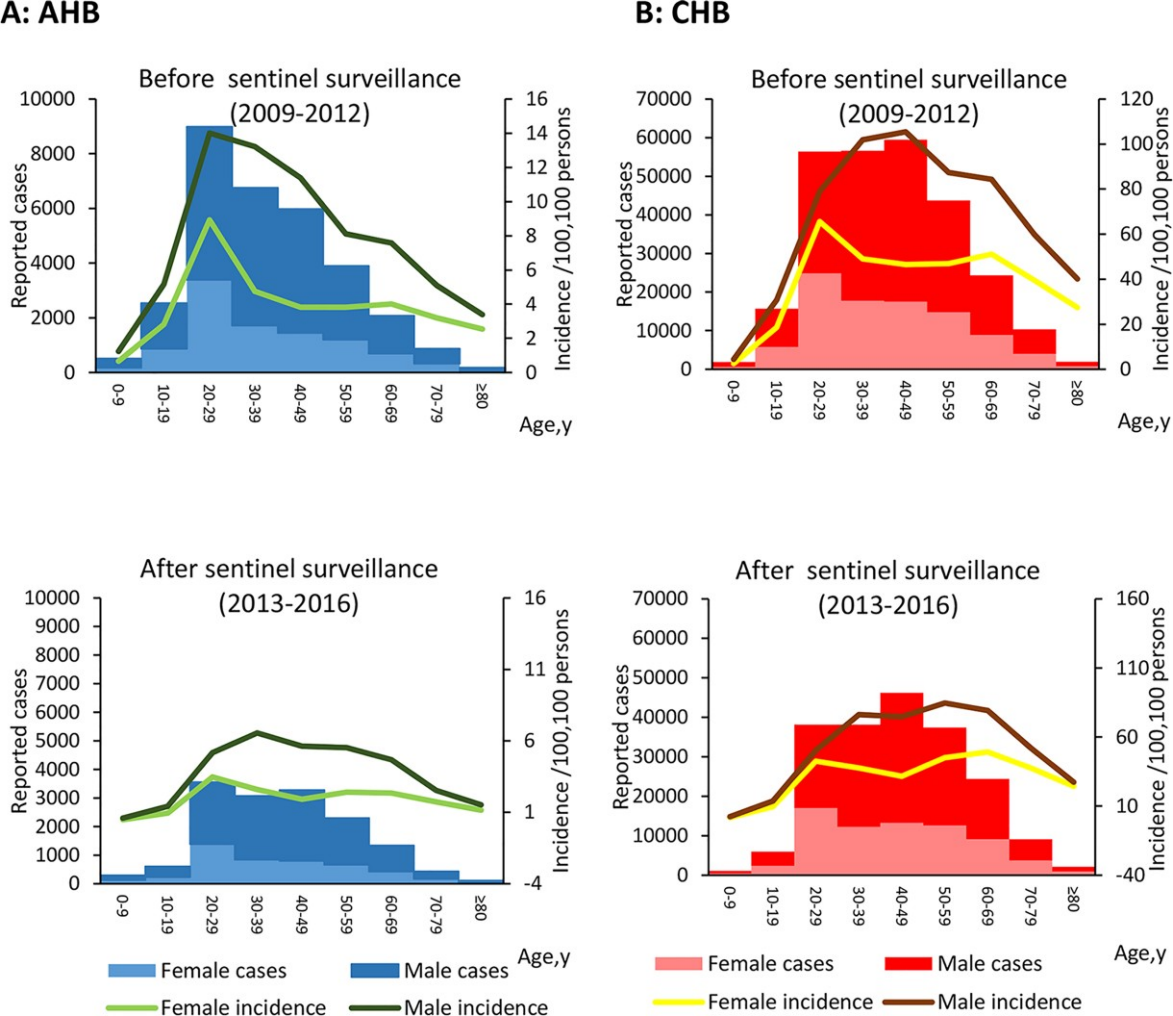
Age-specific and gender-specific cases and incidence of hepatitis B (acute and chronic), China, before and after sentinel surveillance. Age-specific and gender-specific cases and incidence of acute hepatitis B, China, before and after sentinel surveillance. (B) Age-specific and gender-specific cases and incidence of chronic hepatitis B, China, before and after sentinel surveillance. Incidence rates were analyzed by time period (before and after enhancement of surveillance) and age group.

Fig 5 shows the incidence of AHB and CHB by age and gender; the incidence of AHB declined in all age groups after enhancement of surveillance. After enhanced sentinel surveillance, the incidence of AHB among 30–39 year-olds (4.6/100,000) was highest, followed by 20–29 year-olds (4.4/100,000) and 50–59 year-olds (4.0/100,000). The highest CHB incidence was among 50–59 year-olds (65.3/100,000), followed by 60–69 year-olds (64.6/100,000) and 30–39 year-olds (57. 5/100,000) (See Fig 5).

### Positive predictive values

We obtained serum from 5,734 AHB cases reported between 2013 and 2016 (40.4% of reported cases), and tested the sera for IgM anti-HBc. The case reporting PPV in the eastern region was higher than in the central and western regions every year from 2013 to 2016, except 2015. PPVs in different regions increased between 2013 and 2016 (eastern: χ^2^_trend_ = 20.68, P <0.0001, central: χ^2^_trend_ = 105.91, P <0.0001, western: χ^2^_trend_ = 37.88, P <0.0001). PPV in urban areas (69.7%) was higher than in rural areas (45.8%). PPV increased from 2013 to 2016 in both urban and rural areas (Urban: χ^2^_trend_ = 90.62, P <0.0001, Rural: χ^2^_trend_ = 10.69, P = 0.001). PPV among 0–19 year-olds was lower than among ≥20-year-olds between 2013 and 2016 (P<0.001). PPV increased in every age group, except among 0-19-year-olds. No significant difference in PPV was seen between males and females (P>0.05). PPV increased among males and females between 2013 and 2016 (male: χ^2^_trend_ = 97.40, P <0.0001, female: χ^2^_trend_ = 23.09, P <0.0001) (See Table 1).

**Table 1:** PPV of acute hepatitis B diagnosis by region, location type, age group and gender, China, 2013–2016.

Characteristic		2013			2014			2015			2016			Total		
		Reported as acute^1^	PPV	*χ*^*2*^/*P*	Reported as acute	PPV	*χ*^*2*^/*P*	Reported as acute	PPV	*χ*^*2*^/*P*	Reported as acute	PPV	*χ*^*2*^/*P*	Reported as acute	PPV	*χ*^*2*^/*P*
Region																
	Eastern	810	68.6	*χ*^*2*^ = 105.53	668	67.5	*χ*^*2*^ = 58.90	586	74.6	*χ*^*2*^ = 70.54	574	78.6	*χ*^*2*^ = 53.21	2638	71.8	*χ*^*2*^ = 235.90
	Central	407	49.4	*P*<0.001	321	64.2	*P*<0.001	403	86.1	*P*<0.001	200	77.5	*P*<0.001	1331	68.3	*P*<0.001
	Western	560	41.8		451	45.2		281	57.7		473	59.0		1765	49.8	
Location type^2^																
	Urban	1250	62.0	*χ*^*2*^ = 66.36	1122	64.1	*χ*^*2*^ = 38.90	1088	77.9	*χ*^*2*^ = 47.64	961	76.9	*χ*^*2*^ = 71.49	4421	69.7	*χ*^*2*^ = 250.45
	Rural	527	41.0	*P*<0.001	318	44.7	*P*<0.001	182	53.8	*P*<0.001	286	51.0	*P*<0.001	1313	45.8	*P*<0.001
Age group																
	0~19	24	37.5		14	35.7		11	63.6		31	38.7		80	41.3	
	20~39	725	56.3	*χ*^*2*^ = 5.75	561	64.2	*χ*^*2*^ = 10.39	462	76.6	*χ*^*2*^ = 2.78	493	75.3	*χ*^*2*^ = 21.88	2241	66.6	*χ*^*2*^ = 29.82
	40~59	712	57.4	*P* = 0.124	598	58.0	*P* = 0.016	571	74.1	*P* = 0.427	493	70.6	*P*<0.001	2374	64.3	*P*<0.001
	60~	316	52.2		267	55.8		226	71.7		230	67.0		1039	60.6	
Gender																
	Male	1270	54.3	*χ*^*2*^ = 3.73	996	58.8	*χ*^*2*^ = 1.23	952	73.7	*χ*^*2*^ = 1.12	883	70.9	*χ*^*2*^ = 0.008	4101	63.5	*χ*^*2*^ = 3.379
	Female	507	59.4	*P* = 0.053	444	61.9	*P* = 0.268	318	76.7	*P = 0*.*29*	364	71.2	*P* = 0.927	1633	66.1	*P* = 0.066
Total		1777	55.8		1440	59.8		1270	74.5		1247	71.0		5734	64.2	

1 Reported as acute: cases reported as AHB and at the same time tested serum for IgM anti-HBc.

2 Definitions of urban and rural areas are according to the National Bureau of Statistics.

## Discussion

We have shown that enhancing surveillance can reduce the misclassification of reported hepatitis B cases in China by decreasing the proportion of unclassified cased and increasing the diagnostic accuracy for acute hepatitis B virus infection. The positive predictive value of acute hepatitis B reports increased annually when tested against a gold standard that included testing for IgM anti-HBc. Enhancement of surveillance resulted in improved reporting accuracy for all regions, regardless of sex or urban-rural status. Improvement in accuracy was less pronounced for western and rural areas.

The percent of unclassified hepatitis B cases was 4.2% in 2016—a sharp decrease of 87% compared to pre-enhanced surveillance, and a figure that is consistent with the UHB proportion found by Chen and colleagues in Fujian province [10]. Chronic hepatitis B infection can be a silent epidemic, as individuals are often infected when they are young but symptoms only appear decades later[11]. Decades without symptoms facilitates transmission of HBV because asymptomatic individuals with CHB are infectious. China now has a very large population with CHB—nearly 90 million people, according to the most recent national serosurvey, with the vast majority being infected before the availability of hepatitis B vaccine [12]. It can be difficult to distinguish AHB from a flare-up of CHB in a single examination—for example, laboratory tests cannot make the distinction if the history of infections is unknown. The large population with CHB in China, coupled with imperfect diagnostic specificity, challenges the identification of new infections. However, by standardizing the additional history data necessary for diagnosing the stage of infection, enhanced surveillance was able to decrease the proportion of unclassified cases.

Enhanced surveillance was associated with an increased PPV of an AHB report. By 2016, AHB PPV was 71.0%, rising from 55.8%, and ultimately higher than PPV in 8 provinces without enhanced surveillance as seen by Wang and colleagues [6]. WHO recommends PPV as a key characteristic of importance for surveillance systems [13]. High AHB PPV enables public health officials to focus resources more accurately on the prevention of hepatitis B infections. HBV carriers and CHB flare-ups being mistakenly reported as acute infections [14] lowers PPV of AHB reports. For enhancing surveillance, we added the absence of symptoms together with ALT results to serve as a reminder to clinicians that only patients with symptoms should be reported to NNDRS. The further addition of IgM Anti-HBc testing helped to differentiate acute from chronic hepatitis B and minimize the proportion of misclassified cases.

We found that the PPV of AHB reports from eastern and central areas were higher than in western areas. This may be because the laboratory capacity of hospitals in western areas was less than that in eastern and central areas[15] and that the accuracy of reporting cards for NNDRS was lower in the western areas[16, 17]. Greater laboratory capacity in urban areas is likely the reason for a higher PPV in urban areas than in rural areas. We speculate that the reason PPV was lower among 0–19 year-olds than among adults was because the symptoms of the younger people with HBV infection were relatively mild, and the classification of acute and chronic hepatitis B was difficult. With the growth of age, the symptoms of the hepatitis B were more obvious, facilitating the diagnosis of chronic hepatitis B. Zheng's study was consistent with this point[18].

Starting in 2009, inpatients must be screened for hepatitis B and C during hospitalization in China, and HBsAg positive individuals are prohibited from donating blood. We believe that this may have led to the decline by 16.2% in AHB reports that we observed in the pre-enhanced surveillance period. The sharper, 53.7% decline immediately following implementation of enhanced surveillance in these sentinel counties was likely due to the enhancement of surveillance. The rate of decreasing in enhanced surveillance sites(53.7%) was faster than the national level(17.2%)[19].We believe that standardized training and use of a flow chart helped clinicians distinguish acute from chronic hepatitis B, and that the additional required information reminded doctors to be aware of the stage of HBV infection. Including IgM anti-HBc testing in the enhancement of surveillance encouraged hospitals to develop IgM anti-HBc testing capacity, further increasing the accuracy of reported AHB.

The World Health Organization’s Global Health Sector Strategy on Viral Hepatitis [20] aims to eliminate viral hepatitis as a major public health threat by 2030, specifically targeting reduction of the incidence of new hepatitis B infections. Accurate surveillance for hepatitis B infection will therefore be important for assessing progress toward the WHO objectives and for adjusting prevention strategies along the way. By minimizing misreported cases, surveillance data can more accurately reflect the epidemiology of hepatitis B, facilitating outbreak detection and identification of risk factors for new infections to target vaccination strategies.

Our study has strengths and limitations. Strengths are that all 31 provinces in mainland China had enhanced surveillance counties, including urban and rural counties, and that all serum specimens tested with the same method and reagents. A limitation is that only 40% of the enhanced surveillance AHB reports had IgM anti-HBc testing performed. Although 40% is lower than needed for representativeness, it is much higher than the 1.5% IgM anti-HBc testing seen in China without enhancement [7]. A second limitation is that the diagnosis of acute hepatitis B was based on a positive IgM anti-HBc and positive HBsAg together with symptoms related to hepatitis B. However, IgM anti-HBc is present in approximately 10%–15% of patients with chronic hepatitis B, especially in CHB with an acute flare-up [21–23]. This will tend to overestimate AHB PPV for the large number of CHB patients in China. A third weakness is that PPV was only able to be determined in the post-enhancement period, as the determination of PPV relies on specific elements of surveillance enhancement.

In summary, enhanced hepatitis B surveillance was associated with an increased accuracy of AHB diagnoses and with fewer reports of unclassified hepatitis B. Our study supports a recommendation to extend enhanced surveillance nationwide to improve the quality of national hepatitis B surveillance. We also recommend additional effort to raise western and rural areas clinicians’ diagnostic and reporting capacity and to ensure sufficient resources to make hepatitis B stage-specific diagnoses using IgM anti-HBc testing.

## Supporting information

S1 Fig Reported cases and incidence of hepatitis B (acute and chronic) before and after sentinel surveillance, China, 2009–2016. (XLSX)

S2 Fig The proportion of unclassified hepatitis B, China, before and after sentinel surveillance. (XLSX)

S3 Fig Age-specific and gender-specific cases and incidence of hepatitis B (acute and chronic), China, before and after sentinel surveillance. (XLSX)
